# Monitoring Adverse Drug Events in Web Forums: Evaluation of a Pipeline and Use Case Study

**DOI:** 10.2196/46176

**Published:** 2024-06-18

**Authors:** Pierre Karapetiantz, Bissan Audeh, Akram Redjdal, Théophile Tiffet, Cédric Bousquet, Marie-Christine Jaulent

**Affiliations:** 1 Inserm Sorbonne Université université Paris 13, Laboratoire d’informatique médicale et d’ingénierie des connaissances en e-santé, LIMICS, F-75006 Paris France; 2 Service de santé publique et information médicale CHU de Saint Etienne 42000 Saint-Etienne France; 3 Institut National de la Santé et de la Recherche Médicale, Université Jean Monnet, SAnté INgéniérie BIOlogie St-Etienne, SAINBIOSE 42270 Saint-Priest-en-Jarez France

**Keywords:** pharmacovigilance, social media, scraper, natural language processing, signal detection, graphical user interface

## Abstract

**Background:**

To mitigate safety concerns, regulatory agencies must make informed decisions regarding drug usage and adverse drug events (ADEs). The primary pharmacovigilance data stem from spontaneous reports by health care professionals. However, underreporting poses a notable challenge within the current system. Explorations into alternative sources, including electronic patient records and social media, have been undertaken. Nevertheless, social media’s potential remains largely untapped in real-world scenarios.

**Objective:**

The challenge faced by regulatory agencies in using social media is primarily attributed to the absence of suitable tools to support decision makers. An effective tool should enable access to information via a graphical user interface, presenting data in a user-friendly manner rather than in their raw form. This interface should offer various visualization options, empowering users to choose representations that best convey the data and facilitate informed decision-making. Thus, this study aims to assess the potential of integrating social media into pharmacovigilance and enhancing decision-making with this novel data source. To achieve this, our objective was to develop and assess a pipeline that processes data from the extraction of web forum posts to the generation of indicators and alerts within a visual and interactive environment. The goal was to create a user-friendly tool that enables regulatory authorities to make better-informed decisions effectively.

**Methods:**

To enhance pharmacovigilance efforts, we have devised a pipeline comprising 4 distinct modules, each independently editable, aimed at efficiently analyzing health-related French web forums. These modules were (1) web forums’ posts extraction, (2) web forums’ posts annotation, (3) statistics and signal detection algorithm, and (4) a graphical user interface (GUI). We showcase the efficacy of the GUI through an illustrative case study involving the introduction of the new formula of Levothyrox in France. This event led to a surge in reports to the French regulatory authority.

**Results:**

Between January 1, 2017, and February 28, 2021, a total of 2,081,296 posts were extracted from 23 French web forums. These posts contained 437,192 normalized drug-ADE couples, annotated with the Anatomical Therapeutic Chemical (ATC) Classification and Medical Dictionary for Regulatory Activities (MedDRA). The analysis of the Levothyrox new formula revealed a notable pattern. In August 2017, there was a sharp increase in posts related to this medication on social media platforms, which coincided with a substantial uptick in reports submitted by patients to the national regulatory authority during the same period.

**Conclusions:**

We demonstrated that conducting quantitative analysis using the GUI is straightforward and requires no coding. The results aligned with prior research and also offered potential insights into drug-related matters. Our hypothesis received partial confirmation because the final users were not involved in the evaluation process. Further studies, concentrating on ergonomics and the impact on professionals within regulatory agencies, are imperative for future research endeavors. We emphasized the versatility of our approach and the seamless interoperability between different modules over the performance of individual modules. Specifically, the annotation module was integrated early in the development process and could undergo substantial enhancement by leveraging contemporary techniques rooted in the Transformers architecture. Our pipeline holds potential applications in health surveillance by regulatory agencies or pharmaceutical companies, aiding in the identification of safety concerns. Moreover, it could be used by research teams for retrospective analysis of events.

## Introduction

### Social Media as a Complementary Data Source for Pharmacovigilance

One primary mission of regulatory agencies such as the FDA (Food and Drug Administration) or the EMA (European Medicines Agency) is to monitor drug usage and adverse drug events (ADEs) to mitigate the risks associated with drugs within the population. This task entails analyzing diverse data sources, including clinical trials, postmarketing surveillance, spontaneous reporting systems, and published scientific literature. Despite the wealth of available data, some ADEs are not always detected promptly, largely because of underreporting. In France, for instance, underreporting was estimated to range between 78% and 99% from 1997 to 2002 [1]. To tackle this challenge, several countries have implemented systems allowing patients to report ADEs.

Additional sources for detecting ADEs have been under exploration, such as electronic patient records [2-4] and social media platforms [5-9]. While some argue that social media alone cannot serve as a primary source for signal detection [10], it can be viewed as a valuable secondary source for monitoring emerging adverse drug reactions or reinforcing signals previously identified through spontaneous reports stored in traditional pharmacovigilance databases [11]. In a prior study by the authors, patient profiles and reported ADEs found in web forums were compared with those in the French Pharmacovigilance Database (FPVD). The forums tended to represent younger patients, more women, less severe cases, and a higher incidence of psychiatric disorder–related ADEs compared with the FPVD [12]. Moreover, forums reported a greater number of unexpected ADEs. Over the past decade, several tools for evaluating social media posts have been described in the literature [13]. Specifically, effective ADE detection in social media necessitates both quantitative and qualitative analyses of data [14].

### Qualitative Approach for Individual Assessment of Posts

Qualitative assessment entails evaluating whether users’ messages contain pertinent information for an assessment akin to a pharmacovigilance case report. This includes details such as the patient’s age and gender, the severity of the case, the expectedness and timeline of the adverse event, time-to-onset, dechallenge (outcome upon drug withdrawal), and rechallenge (outcome upon drug reintroduction). For instance, GlaxoSmithKline Inc. implemented the qualitative approach Insight Explorer, which facilitates the collection of extensive data for causality and quality assessment. Users can input data including personal information (eg, age range, gender) and product details (eg, name, route of administration, duration of use, dosage). This approach was adapted for the WEB-RADR (Recognizing Adverse Drug Reactions) project to manually construct a gold standard of curated patient-authored text [15].

### Quantitative Approach for Monitoring Adverse Drug Events on Social Media

Quantitative evaluation involves analyzing extracted data using descriptive and analytical statistics, such as signal detection and change-point analysis. Numerous projects have been undertaken to monitor ADEs on social media. One of the earliest projects is the PREDOSE (Prescription Drug Abuse Online Surveillance and Epidemiology) project [5], which investigates the illicit use of pharmaceutical opioids reported in web forums. While the PREDOSE project showcased the potential of leveraging social media for opioid monitoring, notable limitations are the lack of deidentification and signal detection methods. MedWatcher Social, a monitoring platform for health-related web forums, Twitter, and Facebook, represents a prototype application developed in 2014 [16]. Yeleswarapu et al [6] outlined a semiautomatic pipeline that applies natural language processing (NLP) tasks to extract ADEs from MEDLINE abstracts and user comments from health-related websites. However, this pipeline was not intended for routine use.

The Domino’s interface [17], developed in 2018 by the University of Bordeaux in France and funded by the French Medicines Agency (Agence nationale de sécurité du médicament et des produits de santé [ANSM]), was designed to analyze drug misuses in health-related web forums using NLP methods and the summary of product characteristics. Initially tailored for antidepressant drugs, this tool does not primarily focus on ADE surveillance.

Another pipeline, described by Nikfarjam et al in 2019 [7], used a neural network–based named entity recognition system specifically designed for user-generated content in social media. This platform is dedicated to identifying the association of cutaneous ADEs with cancer therapy drugs. The study focused on a selection of drugs and only examined 8 ADEs.

Magge et al [8] described a pipeline aimed at the extraction and normalization of adverse drug mentions on Twitter. Their pipeline consisted of an ADE classifier designed to identify tweets mentioning an ADE, which were then mapped to a MedDRA (Medical Dictionary for Regulatory Activities Terminology) code. However, the normalization process was confined to the ADEs present in the training set. Neither Nikfarjam’s nor Magge’s pipeline provides a graphical user interface.

Some private companies also offer tools for analyzing social media for pharmacovigilance purposes. For instance, the DETECT platform was developed as part of a collaborative project in France by Kappa Santé [18]. This system enabled the labeling of posts with known controlled vocabulary concepts, and signal detection was conducted [19]. Within the scope of this project, Expert System Company implemented BIOPHARMA Navigator to extract web forum posts, while the Luxid Annotation Server provided web services for the automatic annotation of posts.

An important finding from the studies of the last decade is that while regulatory agencies have begun using data sources beyond spontaneous reports, social media has yet to be fully leveraged in real-world settings due to the immaturity of available solutions. Primarily, these solutions are essentially proofs of concept that lack scalability and are challenging for experts to evaluate routinely, primarily due to the absence of a graphical user interface to present information.

Our aim was to assess the potential of integrating social media into pharmacovigilance and enhancing decision-making with this novel data source. To achieve this, our objective was to develop and assess a pipeline that processes data from the extraction of web forum posts to the generation of indicators and alerts within a visual and interactive environment. The goal was to create a user-friendly tool that enables regulatory authorities to make better-informed decisions effectively.

This article presents the design and implementation of our pipeline dedicated to harnessing posts from social media. In addition, we showcase the use of the pipeline through a specific use case, emphasizing the importance of monitoring drugs in social media to better address patients’ expectations.

## Methods

### Overview

The PHARES project (Pharmacovigilance in Social Networks), funded from 2017 to 2019 by the French ANSM, aimed to develop a software suite (a pipeline) enabling pharmacovigilance users to analyze social networks, particularly messages posted on forums. The objective of the pipeline is to facilitate routine use through continuous post extraction and quantitative data analysis from web forums, specifically tailored for the French language.

**Figure 1:**
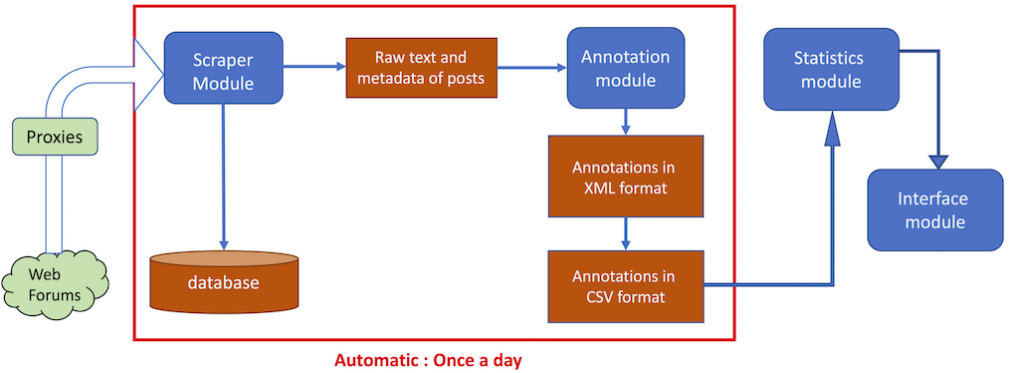
Structure of the PHARES’ pipeline, with modules in blue and data information in red. CSV: comma-separated values; PHARES: Pharmacovigilance in Social Networks.

The pipeline is made up of 4 modules, each referring to its own methods (Figure 1):

The Scraper module, which extracts posts from forums using a previously developed tool, Vigi4Med (V4M) scraper [9], and produces a comma-separated values (CSV) file filled with the texts extracted.

The Annotation module, which extracts elements of interest from the posts and registers annotations in CSV files, with each line representing an annotation of an ADE or a drug. When a causality relationship is identified, both an ADE and a drug are annotated on the same line.

The Statistical module, which performs quantitative analysis on the annotated posts, generating numerical data, tables, or figures.

The Interface module, which supports query definition and visualization of results.

The methodology used to evaluate the PHARES pipeline involved comparing its performance with existing platforms mentioned above, in accordance with a set of criteria established with prospective PHARES users. The criteria, specific to each module, are as follows:

- General level: focus on ADEs, designed for routine usage.
- Scraper: collects all posts of a selected website, performs deidentification, allows to extract posts from web forums, and is open source.
- Statistics: the temporal evolution of posts or annotations is displayed and a change-point analysis (detecting breakpoints) is possible.
- Signal detection: allows to apply at least one signal detection method, displays the temporal evolution of the proportional reporting ratio (PRR), and allows to perform a logistic regression–based signal detection method.
- Graphical user interface: has an interface for users.

### Scraper Module

V4M Scraper is an open-source tool designed for data extraction from web forums [9]. Its primary functions are optimizing scraping time, filtering out posts primarily focused on advertisements, and structuring the extracted data semantically. The module operates by taking a configuration file as input, which contains the URL of the targeted forum. The algorithm navigates through forum pages and generates resource description framework (RDF) triplets for each extracted element, allowing for potential alignment with external semantic resources. A caching mechanism has been integrated into this tool to maintain a local copy of previously visited pages, thereby avoiding redundant requests to websites for already scraped web pages, particularly in cases of errors or testing, for example. Vigi4Med V4M Scraper was customized for the PHARES project, as indicated by the red elements in Figure S1 in Multimedia Appendix 1. The database format (Figure S2 in Multimedia Appendix 1) was implemented to enhance interaction with the interface. Specifically, the main scraping script was adjusted to produce a simplified tabular format (CSV) of the extracted data and to store these data in a database. This modification aims to facilitate input to the subsequent module of the pipeline (annotation). V4M Scraper was customized to enable a continuous scraping routine, wherein data extracted from web forums are automatically and regularly annotated and registered. A log file was integrated into the scraper structure to maintain a record of the last scraped element. This log file ensures that the daily routine scraping always begins from the last scraped point. An automation tool (crontab) is used to schedule the execution of the pipeline for each forum on a daily basis at a specific time.

A total of 23 public French health-related web forums were selected through a combination of Google searches and from a list of certified health websites provided by the HON Foundation, in collaboration with the French National Health Authority (HAS). The selection criteria included the requirement for websites to be hosted in France, feature a discussion board or space for sharing experiences, and have more than 10 patient contributions. Furthermore, Twitter posts are collected and analyzed by the pipeline. This is achieved using the Twitter API for data collection, followed by employing the same modules used for processing web forum posts.

### Annotation Module

Entities corresponding to drugs and pathological conditions in social media were identified and annotated using an NLP pipeline [20]. Initially, conditional random fields were used to account for global dependencies [21]. Specifically, the model considers the entire sequence when making predictions for individual tokens. This approach is advantageous for entity extraction tasks, as the presence of an entity in one part of the text can influence the likelihood of other entities in the vicinity. Second, a support vector machine is used to predict the causality relationship between an entity identified as a drug and another entity identified as an ADE. The annotation method used in this module was implemented at an early stage of the pipeline’s design. Currently, the named entity recognition task of this module is undergoing revision to incorporate more recent advancements in NLP algorithms [22-26].

In a third step, the detected annotations were normalized using codes from the MedDRA and the Anatomical Therapeutic Classification (ATC) to ensure they were suitable for signal detection purposes.

MedDRA is an international medical hierarchical terminology comprising 5 levels used to code potential ADEs in pharmacovigilance. The highest level is the system organ class, which is further divided into high-level group terms, then into high-level terms, preferred terms (PTs), and finally lowest level terms. Typically, the PT level is used in pharmacovigilance signal detection.

The ATC classification system is a drug classification used in France for pharmacovigilance purposes. It categorizes the active ingredients of drugs based on the organ system they primarily affect. The classification comprises 5 levels: the anatomical main group (consisting of 14 main groups), the therapeutic subgroup, the therapeutic/pharmacological subgroup, the chemical/therapeutic/pharmacological subgroup, and the chemical substance. Typically, the fifth level (chemical substance) is used in pharmacovigilance signal detection.

The outputs of the annotation module are CSV files with the following variables:

- Concerning the post: forum name, post ID, and date
- Concerning the ADE: verbatim, normalized term, unified medical language system’s concept unique identifier, and MedDRA code
- Concerning the drug: verbatim, normalized term, active ingredient, and ATC code

In these CSV files, each line can consist of either an adverse event (ADE) annotation, a drug annotation, or both when a causality relationship has been identified between the drug and the ADE. Table 1 provides a sample of the database.

In a prior study, we selected posts where at least one ADE associated with 6 drugs (agomelatine, baclofen, duloxetine, exenatide, strontium ranelate, and tetrazepam) had been detected by this algorithm. A manual review revealed that among 5149 posts, 1284 (24.94%) were validated as pharmacovigilance cases [12]. The fundamental metrics used to assess the performance of the annotation module were precision (P), recall (R), and their harmonic mean *F*_1_-score. To calculate these metrics, it is necessary to evaluate false negatives for nonrecognition of relevant terms, false positives for irrelevant recognitions, and true positives for correct recognitions. Precision, recall, and *F*_1_-score are defined as follows:

Precision = (true positive)/(true positive + false positive); recall = (true positive)/(true positive + false negative); *F*_1_-score = (2 × precision × recall)/(precision + recall) **(1)**

In the “Results” section, we present a comparison of the performance of the annotation module with the performance of state-of-the-art methods [8,22,25,26].

**Table 1:** Sample of the database after annotation and normalization; 8 lines corresponding to 8 annotated couples in the same post.

Forum name	Post ID	Date	Time	ADE^a^ verbatim	ADE normalized	Concept unique identifier	Drug verbatim	Drug normalized	Active ingredient	MedDRA^b^ code	ATC^c^ code
Atoute	7354	October 8, 2018	21:37:00	Maux de tête	Céphalée	C0018681	Lévothyrox	LEVOTHYROX	Levothyroxine sodique	—^d^	H03AA01
Atoute	7354	October 8, 2018	21:37:00	Maux de tête	Céphalée	C0018681	Calcium	—	—	—	—
Atoute	7354	October 8, 2018	21:37:00	Nodules cancereux	—	—	Lévothyrox	LEVOTHYROX	Levothyroxine sodique	—	H03AA01
Atoute	7354	October 8, 2018	21:37:00	Nodules cancereux	—	—	Calcium	—	—	—	—
Atoute	7354	October 8, 2018	21:37:00	Fatigue	Fatigue	C0015672	Lévothyrox	LEVOTHYROX	Levothyroxine sodique	10016256	H03AA01
Atoute	7354	October 8, 2018	21:37:00	fatigue	Fatigue	C0015672	Calcium	—	—	10016256	—
Atoute	7354	October 8, 2018	21:37:00	Perte de poids	Poids diminué	C0043096	Lévothyrox	LEVOTHYROX	Levothyroxine sodique	10048061	H03AA01
Atoute	7354	October 8, 2018	21:37:00	Perte de poids	Poids diminué	C0043096	Calcium	—	—	10048061	—

^a^ADE: adverse event.

^b^MedDRA: Medical Dictionary for Regulatory Activities Terminology.

^c^ATC: Anatomical Therapeutic Classification.

^d^No data are available for this slot.

### Statistical Module

This module generates general statistics and diagrams for web forums or Twitter. It provides data such as the number of annotated posts (related to the drug, the ADE, or both), the count of drug-ADE pairs identified, and the distribution of ADEs’ MedDRA-PTs. In addition, a change-point analysis method was used to detect significant changes over time in the mean number of posts mentioning the drug and ADE [27].

Besides, several statistical signal detection methods were implemented to generate potential signals. Safety signals, which provide information on adverse events that may potentially be caused by a medicine, were further evaluated by pharmacovigilance experts to determine the causal relationship between the medicine and the reported adverse event.

The statistical module implements 3 signal detection methods, including 2 well-known and frequently used disproportionality signal detection methods: the PRR [28] and the reporting odds ratio (ROR) [29]. In addition, a complementary method, a logistic regression–based signal detection method known as the class imbalanced subsampling lasso [30], was used.

PRR and ROR are akin to a relative risk and an odds ratio, respectively. However, they differ in their denominators: as the number of exposed patients is typically unknown in pharmacovigilance databases, the denominator in PRR and ROR calculations is the number of cases reported in the pharmacovigilance database.

PRR and ROR are specific to each drug-ADE pair and can be directly computed from the contingency table (Table 2).

**Table 2:** Contingency table for disproportionality analysis.

	Adverse drug event of interest	Other adverse drug events
Drug of interest	*A*	*b*
Other drugs	*C*	*d*

The PRR compares the proportion of an ADE among all the ADEs reported for a specific drug with the same proportion for all other drugs in the database (Equation 2). A PRR significantly greater than 1 suggests that the ADE is more frequently reported for patients taking the drug of interest, while a PRR equal to 1 suggests independence between the 2 variables.

PRR = [a/(a + b)]/[c/(c + d)] **(2)**

The ROR quantifies the strength of the association between drug administration and the occurrence of the ADE. It represents the ratio of the odds of drug administration when the ADE is present to the odds of drug administration when the ADE is absent (Equation 3). When the 2 events are independent, the ROR equals 1. An ROR significantly greater than 1 suggests that drug administration is associated with the presence of the ADE.

ROR = *ad*/*bc*
**(3)**

We considered events over posts for the calculation of disproportionality statistics. If the same drug-ADE pair was identified multiple times within a post, the pair was counted as many times as it occurred in the calculation.

Disproportionality analysis has certain limitations, including the confounding effect resulting from coreported drugs and the masking effect, where the background relative reporting rate of an ADE is distorted by extensive reporting on the ADE with a specific drug or drug group. Caster et al [31] demonstrated through 2 real case examples how multivariate regression–based approaches can address these issues. Harpaz et al also suggested that logistic regression could be used for safety surveillance [32]. Initially designed for pharmacovigilance case reports, we hypothesize that they may also be applicable to posts.

The logistic regression model specifically focuses on a particular ADE or a group of ADEs. It involves creating a vector that represents the presence (1) or absence (0) of the ADE of interest in the pharmacovigilance case (in our case, in the post). Additionally, a matrix is generated to represent the administration or nonadministration of all drugs in the database by the patient (1 for administration and 0 for nonadministration). Figure S3 in Multimedia Appendix 1 illustrates an example of using logistic regression. In our case, we assumed that if a drug was annotated in the post, it was taken by the patient. The logistic regression aims to predict the probability of the presence of the ADE (ADE=1) of interest based on the presence of all (*N_m_*) drugs in the database (Equation 4), where *X* represents the distribution of the presence/absence of the drugs. The adjusted factors included only concomitant medications, as patient-related factors are often missing in web forums’ posts. Therefore, we did not need to address the impact of missing data, which should be evaluated when necessary.

ln([P(X|ADE=1)]/[P(X|ADE=0)]) = a + b1 × Drug1 + ^...^ + bi × Drug_i_ + ^.. .^+ bNm × Drug_Nm_
**(4)**

The selection of the drugs depends on the parameter *b_i_*. If *b_i_*<0, the drug *i* decreases the risk of the ADE, and if *b_i_*>0, the drug *i* increases the risk of the ADE.

Then, 2 sets are defined:

- *S*_1_: set of *n*_1_ posts with an annotation of the ADEs of interest.
- *S*_0_: set of *n*_0_ posts without an annotation of the ADEs of interest.

In our case *n*_0_>>*n*_1_, indicating a significant imbalance toward posts lacking annotations of the ADEs of interest. To address this issue, we took a subsample with a more favorable ratio of posts with annotated ADEs versus those without. Additionally, to enhance result stability, we conducted multiple draws instead of just one.

In practice, we generated *B* subsamples. Each subsample was constructed by randomly drawing, with replacement, *n*_1_ posts from *S*_1_ and R posts from *S*_0_, where R=max(4*n*_1_, 4*N_m_*). The choice of 4*n*_1_ was inspired by case-control studies, while 4*N_m_* was included to ensure an adequate number of observations considering the multitude of predictors.

The maximum number of drug predictors is set to 50 and the method is then applied on *B*=250 drawings. Finally, the distribution of interest is the distribution of the number of times the drug was selected as a predictor (*b_i_*>0). The drugs retained as final predictors are those in which the 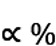 quantile of this distribution is superior to 0. 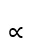 can be equal to 5, 10, or 15.

We implemented a change-point analysis method described in [27] to detect whether there was a change in the evolution over time of a chosen statistic, such as the number of a specific drug-ADE pair, the number of ADEs associated with a specific drug, or the number of drugs associated with a specific ADE. The method uses the Cumulative Sum (CUSUM) algorithm to analyze the evolution of statistics over time, comparing current values with the period mean. It identifies breakpoints by calculating the highest difference in statistical values and comparing it with random samples. The process repeats for periods before and after detected breakpoints until no more are found.

### User Interface Module

**Figure 2:**
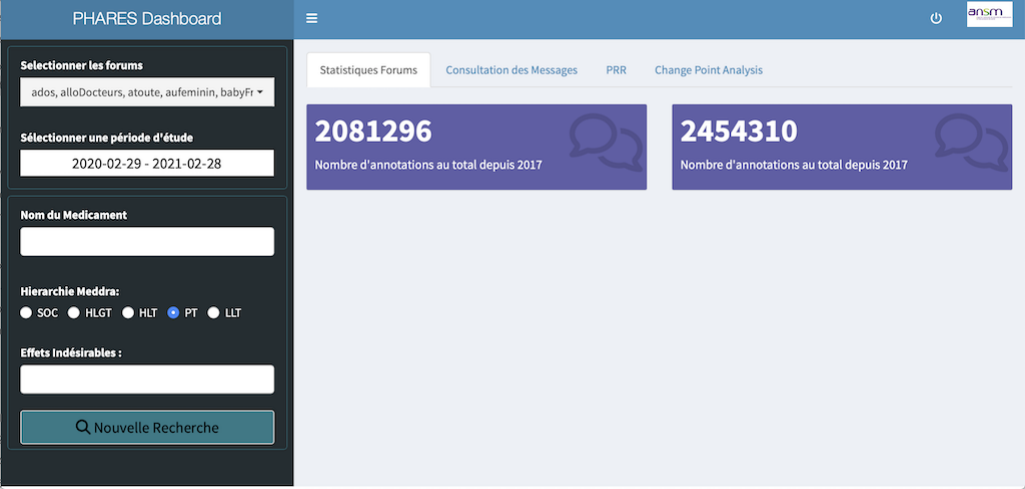
Home of the interface before a query. The section on the left allows to perform a query, while the central section shows the total number of annotated posts since 2017 (n=2,081,296) and the total number of annotations since 2017 (2,454,310).

The user interface module facilitates user interaction with the pipeline in a user-friendly manner. The interface comprises a dashboard divided into 2 main parts. The left dark column (Figure 2) serves as a control sidebar, where users can select parameters to filter the data, including the forum, period, drug(s) according to the ATC classification, and ADE(s) according to a level in the MedDRA hierarchy. On the right side of the interface, various visualizations are available, organized into several tabs such as “Forum Statistics” and “Consultation of Posts,” with additional tabs for statistics that become active upon querying.

Before applying a specific query, the interface provides general information about the currently available data (Figure 2), including the total annotated posts since 2017 (n=2,081,296) and total annotations since 2017 (n=2,454,310). In addition, a “Consultation of Tweets” tab (not visible in the figure) displays the total annotated tweets since March 2020 (n=46,153).

**Figure 3:**
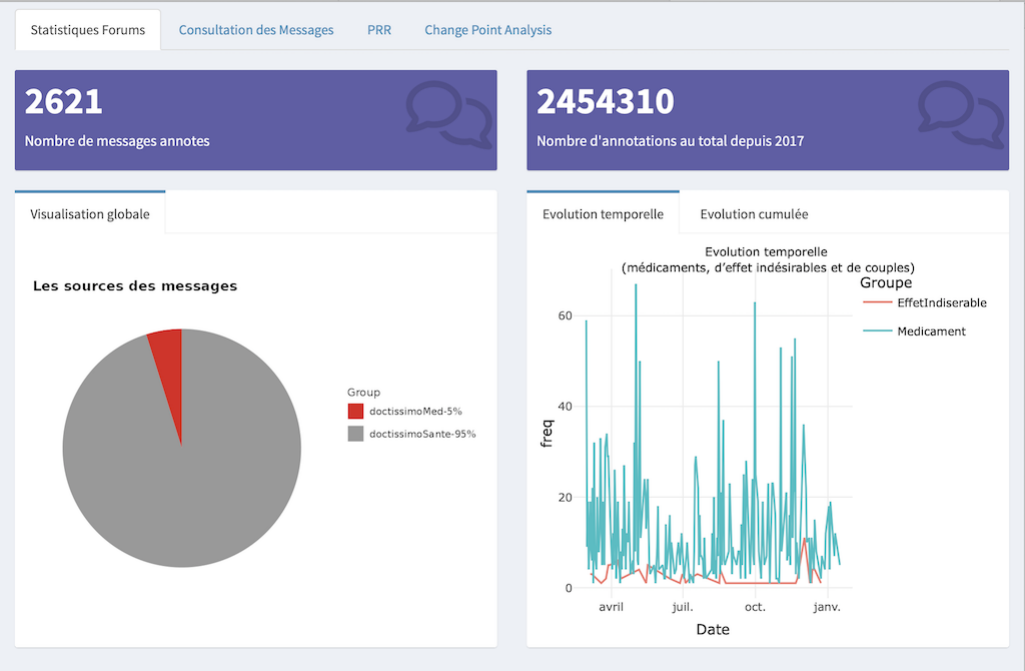
The Statistic forum tab displaying the central section's upper part after a query (Paracetamol and dizziness). The image displays the number of posts related to the selected couple, the number of times the selected couples is annotated, a pie chart with the distribution of web forums, and a line chart with the evolution of the mentions of the drug, the ADE, and the couple. ADE: adverse drug event.

**Figure 4:**
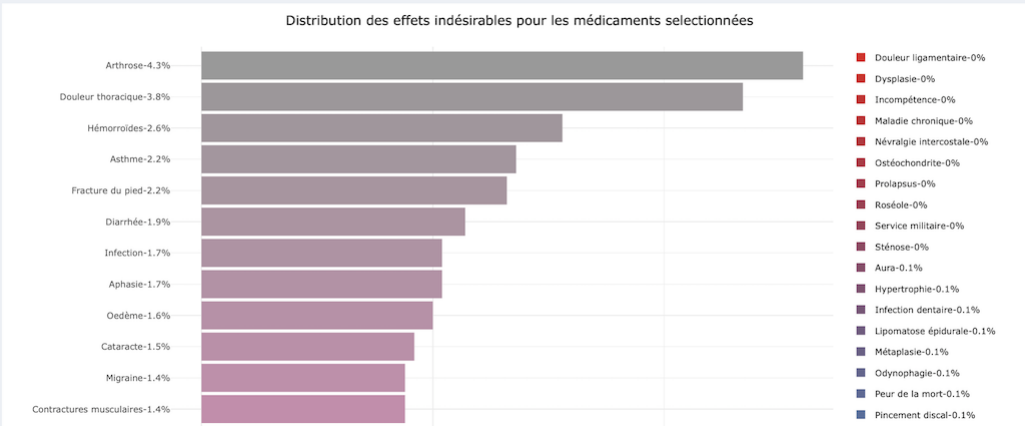
The Statistic forum tab showing the central section's lower part after a query (Paracetamol and dizziness). The image displays an histogram of the distribution of ADEs grouped under "Preferred Terms." ADE: adverse drug event.

Furthermore, several tabs corresponding to different types of statistics, including “Forums Statistics” and “Twitter Statistics,” provide general statistics and diagrams for web forums and Twitter. Examples of these are pie charts showing forum distribution, line charts depicting the evolution of drug and ADE mentions, histograms displaying ADE distribution by system organ class, and line charts illustrating the temporal trend of posts containing the drug and an ADE, as shown in Figures 3 and 4. The “Annotations Plot” tab displays annotations of drugs and adverse effects selected by the user, along with forum information, PTs, high-level terms, high-level group terms, dates, and hours. The “Logistic Regression” tab allows users to choose parameters for applying logistic regression. In the “Disproportionality” tab, users can choose between the PRR and ROR methods, with the time evolution of the chosen method displayed. The “Change-Point” tab enables analysis of temporal evolution, with identified breakpoints indicated. The “Consultation of Posts” and “Consultation of Tweets” tabs provide details on annotated posts/tweets, including downloadable tables. The statistical module performs calculations based on user queries, updating the interface accordingly. If multiple drugs or adverse events are selected, they are treated as new entities for analysis.

The interface was implemented using the R language and environment (R Foundation) for statistical computing and graphics [33], leveraging the Shiny package [34] for development.

### Ethical Considerations

A statement by an Institutional Review Board was not required because we used only publicly available data that do not necessitate Institutional Review Board review.

This study complied with the European General Data Protection Regulation (GDPR), which has been in force since 2018 in Europe [35]. The GDPR enhances the protection of individuals by introducing the right to be informed about the processing of personal data. However, informing each user individually may be impractical. Therefore, the GDPR introduces 2 legal conditions where informed consent is not mandatory, which can be interpreted as supporting the processing of web forum posts for pharmacovigilance (Article 9): “(e) processing relates to personal data which are manifestly made public by the data subject; [. . .] (i) processing is necessary for reasons of public interest in the area of public health, such as [. . .] ensuring high standards of quality and safety of health care and of medicinal products . . ..” The GDPR also requires data processing to “not permit or no longer permits the identification of data subjects” (Article 89). Deidentification was conducted during the extraction of posts from web forums to ensure privacy [9]. User identifiers in the main RDF file were encrypted using the SHA1 algorithm [36]. The correspondence between these encrypted identifiers and the original keys is presented in RDF triplets in a separate file, referred to as the “keys file.” Therefore, the only way to retrieve the original authors’ identities is by concatenating the main RDF containing the encrypted data with the keys file, which is kept in a secured location. Moreover, all our data processing was carried out on a secured server with restricted access.

## Results

### General Results About the Pipeline

The primary outcome of this study is the operational PHARES pipeline itself. Daily extraction and annotation of posts are initiated and imported into the database linked to the user interface. In this paper, the platform’s use will be demonstrated through a specific use case on the analysis of Levothyrox ADE mentions in forums (discussed later). In addition, we conducted a comparative analysis of the PHARES pipeline with the existing platforms mentioned in the “Introduction” section, based on the criteria listed in the “Methods” section.

Of the 10 identified pipelines, half were public and half were private. While 8 out of 10 focused on ADEs, only 4 were designed for routine usage. Five scrapers were open source, and all posts from considered websites were extracted by only 6 of the scrapers (with others extracting posts under certain conditions). Six scraped web forum posts, but only 3 performed deidentification. Additionally, 4 pipelines focused on the French language. A total of 6 pipelines displayed the temporal evolution of the number of posts, but only 1 conducted a change-point analysis. Signal detection methods were performed by only 4 of them, with none displaying the temporal evolution of the PRR nor a logistic regression–based method. Finally, 6 of them had an interface (Table 3).

**Table 3:** PHARES^a^ and identified pipelines’ characteristics match with the identified evaluation criteria^b^.

Pipeline	General			Scraper					Annotation		Statistics			Signal detection			
	Focus on ADEs^c^	Routine usage	Public/private	All posts	Deidentification	Web forums	Open source	French language		Temporal evolution		Change-point analysis	Signal detection		PRR^d^ temporal evolution	Logistic regression	Interface
PREDOSE^e^	X	✓	Public	✓	X	✓	✓	X		✓		X	X		X	X	✓
Insight Explorer	✓	X	Private	X	X	X	✓	X		X		X	X		X	X	✓
MedWatcher Social	✓	✓	Public	X	X	✓	✓	X		✓		X	✓		X	X	✓
Yeleswarapu et al [6]	✓	X	Private	X	X	X	X	X		X		X	✓		X	X	X
Domino	X	✓	Public	✓	X	✓	✓	✓		✓		X	X		X	X	✓
Nikfarjam et al [7]	✓	X	Public and Private	X	X	X	X	X		X		X	X		X	X	X
Magge et al [8]	✓	X	Public	✓	X	X	✓	X		✓		X	X		X	X	X
ADR-PRISM^f^	✓	X	Public and Private	✓	✓	✓	X	✓		✓		X	✓		X	X	✓
Kappa Santé	✓	✓	Private	✓	✓	✓	X	✓		✓		✓	✓		X	X	✓
Expert System	✓	X	Private	✓	✓	✓	X	✓		X		X	X		X	X	✓

^a^PHARES: Pharmacovigilance in Social Networks.

^b^The X symbol means that the characteristic is missing and the symbol ✓ means the characteristic is fulfilled.

^c^ADE: adverse drug event.

^d^PRR: proportional reporting ratio.

^e^PREDOSE: Prescription Drug Abuse Online Surveillance and Epidemiology.

^f^ADR-PRISM: Adverse Drug Reaction from Patient Reports in Social Media.

### Annotation Module’s Comparison With Up-to-Date State-of-the-Art Methods

We also compared the performance of our annotation process with those of up-to-date state-of-the-art methods (Table 4).

While the annotation module demonstrated good performance for named entity recognition (*F*_1_-score=0.886), it remains slightly below the state of the art. Presently, in medical texts, the best performances are achieved by Hussain et al [25] and Ding et al [26] for the named entity recognition task, and by Xia [22] for the relationship extraction task. On Twitter, known for its notably more complex data, Hussain et al [25] achieved slightly better results than our annotator, while Ding et al [26] achieved slightly worse results.

**Table 4:** Comparison of our annotation process’ performances with up-to-date state-of-the-art methods. Performances are given as precision, recall, and F1-score and are divided into 2 categories^a^.

Annotator	Language	Data	Natural language processing method	Named entity recognition (precision; recall; *F*_1_-score)	Relationship extraction (precision; recall; *F*_1_-score)
PHARES^b^	French	Patient’s web drug review	Conditional random fields and support vector machines	0.926; 0.845; 0.886	0.683; 0.956; 0.797
Magge et al [8]	English	Twitter	BERT^c^ neural networks	0.82; 0.76; 0.78	—^d^
Xia [22]	English	Medical texts	HAMLE^e^ model	—	0.929; 0.914; 0.921
Hussain et al [25]	English	Medical texts (PubMed) and Twitter	BERT	0.982; 0.964; 0.976 (PubMed) and 0.840; 0.861; 0.896 (X/Twitter)	—
Ding et al [26]	English	Medical texts (PubMed) and Twitter	BGRU^f^ + char LSTM^g^ attention + auxiliary classifier	0.867; 0.948; 0.906 (PubMed) and 0.785; 0.914; 0.844 (Twitter)	—

^a^The 2 categories are entity recognition, which is the detection of a drug or ADE mention, and relationship extraction, which is the detection of a relation between a drug and an ADE.

^b^PHARES: Pharmacovigilance in Social Networks.

^c^BERT: Bidirectional Encoder Representations from Transformer.

^d^Not available.

^e^HAMLE: Historical Awareness Multi-Level Embedding.

^f^BGRU: Bidirectional Gated Recurrent Unit.

^g^LSTM: Long-Short-Term-Memory.

### Summary of the Result

From January 1, 2017, to February 28, 2021, a total of 2,081,296 posts were extracted from 23 French web forums (Table 5). We obtained 713,057 normalized annotations of drugs, 1,527,004 normalized annotations of ADEs, and 437,192 annotations of normalized drug-ADE couples. The number of posts annotated with at least one normalized drug-ADE couple was equal to 125,279 (6.02%). Table 4 summarizes the number of posts extracted per forum, the publication dates, and the description of the web forum. For 1 forum, the publication dates were not available. A total of 9 were generalist health forums, 3 were specialized for parents of a young baby, 2 for families, 3 for mothers, 2 specialized in thyroid issues, 1 for pregnant women, 1 for women, 1 for parents of a teenager or for teenagers, 1 for sports persons, and 1 specialized in rare diseases.

**Table 5:** Number of extracted posts per forum, publication dates of the first and last extracted posts, and forums’ descriptions.

Forum	Extracted posts, n	Publication date of the first extracted post	Publication date of the last extracted post	Description
thyroideNEW	451,253	February 15, 2001	February 25, 2021	Specialized in thyroid issues
doctissimoSante	248,691	March 19, 2003	January 16, 2021	Generalist health forum
doctissimoNutrition	183,730	December 30, 2002	January 16, 2021	Specialized in nutrition
infoBebe	127,341	November 30, 2000	March 08, 2019	Specialized for parents of a young baby
atoute	118,415	February 05, 2005	February 28, 2021	Generalist health forum
notreFamille	97,098	March 16, 2000	October 26, 2017	Specialized for families
magicMaman	96,713	June 14, 1999	February 22, 2021	Specialized for mothers
doctissimoMed	95,531	August 05, 2002	January 15, 2021	Generalist health forum
doctissimoGrossesse	93,449	November 09, 2006	January 15, 2021	Specialized for pregnant women
thyroide	73,376	September 25, 2001	January 07, 2019	Specialized in thyroid issues
aufeminin	72,732	April 05, 2001	January 09, 2020	Specialized for women
mamanVie	69,167	June 07, 2006	April 10, 2019	Specialized for mothers
onmeda	61,428	July 25, 2001	February 24, 2021	Generalist health forum
ados	58,181	June 20, 2006	March 08, 2019	Specialized for parents of a teenager or for teenagers
carenity	52,659	May 16, 2011	August 29, 2020	Generalist health forum
famili	51,844	November 06, 2000	November 17, 2019	Specialized for families
babyFrance	43,806	January 20, 2003	April 30, 2018	Specialized for parents of young baby
bebeMaman	38,450	—^a^	—	Specialized for mothers of young baby
alloDocteurs	15,833	June 15, 2009	February 09, 2021	Generalist health forum
reboot	9383	May 04, 2016	February 25, 2021	Generalist health forum
futura	6765	May 12, 2003	February 22, 2021	Generalist health forum
sportSante	6350	May 10, 2011	January 14, 2020	Specialized for sportsperson
maladieRares	4827	October 09, 2012	May 14, 2020	Specialized in rare diseases
queChoisir	4250	June 16, 2003	February 11, 2021	Generalist health forum

^a^Not available.

### Use Case: Analysis of Levothyrox ADE Mentions in Forums

To demonstrate the usage of the pipeline, we chose to focus on Levothyrox as a case study. Levothyrox is a drug prescribed in France since 1980 for hypothyroidism and circumstances where it is necessary to limit the thyroid-stimulating hormone. In 2017, a new formula of Levothyrox, differing from the 30-year-old drug at the excipient level (with lactose being replaced by mannitol and citric acid in the new formula), was marketed with widespread media coverage. In parallel, an unexpected increase in notifications of ADEs for this drug was detected. Viard et al [37] were unable to find any pharmacological rationale to explain that signal. Approximately 32,000 adverse effects were reported by patients in France in 2017, representing 42% of all the ADEs collected yearly [38]. Most of these notifications concerned the new formulation of Levothyrox and led to the “French Levothyrox crisis.” In 2017, 1664 notifications of ADEs were spontaneously reported by patients to the Pharmacovigilance Center of Nice. Among the 1544 reviewed notifications, 1372 concerned Levothyrox while only 172 concerned other drugs [37].

In this use case, the study period was from January 1, 2017, to February 28, 2021, and the drugs included were 2 drugs from the “H03AA Thyroid hormones” ATC class: “Levothyroxine sodium” and “associations of levothyroxine and liothyronine.” A total of 17 forums were selected as they included at least one post with information about these drugs. Posts were extracted, annotated, and analyzed through the pipeline from several forums (Table 6). Signal detection methods were applied to an ADE chosen as it frequently appeared with Levothyrox in our data: “tiredness.” A signal can be detected when the lower bound of the 95% CI of the logarithm of the PRR is greater than 0. For logistic regression, we applied the tenth quantile. A total of 11,340 posts contained an annotation concerning the drugs of interest. Figure S4 in Multimedia Appendix 1 illustrates the source and evolution over time of these posts. Out of a total of 50,127 annotations of Levothyrox, they principally originated from the Vivre sans thyroïde forum and were mostly posted in mid-2017 (Figure 4, Table 6). The results of the statistical analysis were displayed by the user interface.

ADEs annotated with Levothyrox were mainly from system organ classes: general disorders and administration site conditions (29.6%), metabolism and nutrition disorders (11.6%), and endocrine disorders (11.4%). The PTs mostly found in association with Levothyrox are listed in Table 7. All this information is accessible in the interface module (Figure S5 in Multimedia Appendix 1).

We chose the PT “tiredness” for the signal detection analysis. A total of 85,976 posts were annotated with either one of the drugs of interest or the ADE tiredness. Among them, 1841 Levothyrox-tiredness couples were found, mostly in 2017 (Table 7).

**Figure 5:**
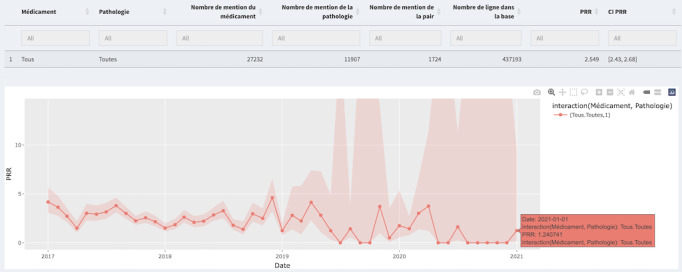
Evolution of the monthly cumulated PRR over time for the annotated couples of Levothyrox-tiredness from 11,340 French web forum posts from January 1, 2017, to February 28, 2021. PRR: proportional reporting ratio.

Figure 5 illustrates the time evolution of the PRR for the Levothyrox-tiredness couple. Figure S6 in Multimedia Appendix 1 displays the source and evolution over time of French web forums’ posts for this couple. A signal is consistently generated throughout the period as the logarithm of the PRR is always greater than 0.

**Table 6:** Number of annotations of Levothyrox per forum in 11,340 posts from French web forums from January 1, 2017, to February 28, 2021.

Forum	Value, n	Cumulative frequency, %
Vivre sans thyroïde	41,211	82.21
Doctissimo Santé	4230	90.65
Doctissimo Grossesse	1476	93.60
Doctissimo Nutrition	1177	95.94
Carenity	863	97.67
Allo docteurs	502	98.67
Atoute	170	99.01
Doctissimo medicaments	166	99.34
Que choisir	85	99.51
Maladie rares	76	99.66
Au feminin	58	99.77
Sport santé	50	99.87
Onmeda	48	99.97
Famili	7	99.98
Futura	5	99.99
Maman vie	2	100.00
Magic maman	1	100.00

**Table 7:** The 20 preferred terms most frequently found with Levothyrox in 11,340 posts from French web forums from January 1, 2017, to February 28, 2021.

Preferred terms	Values, n
Pain	1882
Tiredness	1841
Faintness	1267
Hypothyroidism	1110
Dizziness	912
Insomnia	627
Palpitations	571
Hyperthyroidism	568
Malignant tumor	560
Anxiety	498
Overdose	490
Nervous tension	484
Myalgia	409
Nausea	388
Stress	380
Diarrhea	354
Tachycardia	322
Muscle spasms	321
Convulsions	302
Arthralgia	276

A total of 11 drugs were found to be associated with tiredness using logistic regression: paclitaxel, pegfilgrastim, Levothyrox, glatiramer acetate, escitalopram ferrous sulfate, the combination of Levothyrox and liothyronine, secukinumab, methotrexate, bismuth potassium, tetracycline, and metronidazole.

**Figure 6:**
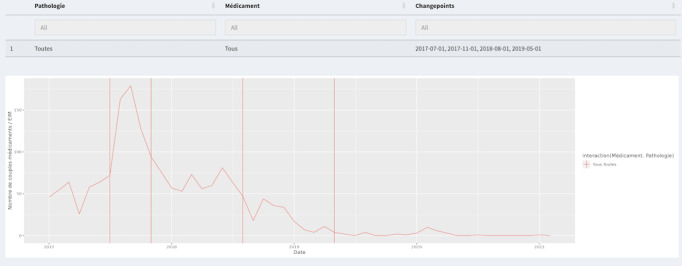
Breakpoints identified with the CPA method (vertical red lines) in the temporal evolution of the number of Levothyrox-ADE couples (horizontal red line) annotated in 11,340 French web forums posts from January 1, 2017, to February 28, 2021. ADE: adverse drug event; CPA: change-point analysis.

Change-point analysis was conducted on the monthly evolution of the number of Levothyrox-ADE couples detected in web forums. Six breakpoints were identified (Figure 6), and 3 of them correlated with an increase in the number of ADEs found with Levothyrox on web forums. These increases occurred in August 2017 and in September and December 2018.

This use case demonstrates that the results obtained through the pipeline, particularly in the context of Levothyrox, align with findings in the literature derived from more traditional data sources such as case reports in pharmacovigilance (see the “Discussion” section). It underscores the potential of leveraging such a pipeline to monitor a drug, not only retrospectively but also in real time using social media. Consequently, PHARES has the capability to potentially uncover new signals in pharmacovigilance.

## Discussion

### Principal Findings

To align with our objective, we implemented and evaluated a pipeline that processes data from the extraction of web forum posts to the generation of indicators and alerts within a visual and interactive environment. Through this pipeline, we demonstrated that quantitative analysis can be conducted through the interface without requiring the user to code. We discovered the feasibility of acquiring information akin to the literature regarding a drug’s ADEs, as well as unexpected ADEs and significant event dates related to a drug. This underscores the relevance and utility of such a pipeline.

A conceptual contribution of this research was the proposal of a methodology for designing a pipeline to facilitate pharmacovigilance studies on web forums. This involved describing 4 independent modules and outlining their interactions. Additionally, another contribution was the adaptation of certain pharmacovigilance analysis methods for the examination of data extracted from web forum posts. The logistic regression–based method presented in this article was originally tailored for pharmacovigilance cases to consider co-prescriptions of drugs. We have adapted it to suit the analysis of pharmacovigilance data extracted from web forum posts.

### Comparison With Prior Work

The PHARES pipeline offers added value compared with previous pipelines in terms of the criteria set, which reflects an analysis of experts’ needs for routine monitoring of ADEs in social media. Unlike previous approaches, the scrapers used in PHARES routinely perform deidentification, and the inclusion of change-point analysis, the evolution of PRRs over time, and a logistic regression–based signal detection method were previously unavailable. The temporal evolution of the number of posts and a signal detection method are also seldom supported. Designed for routine usage and focused on ADEs, all posts from selected web forums are scraped and deidentified using an open-source scraper.

The period and selected web forums differed between both studies: Audeh et al [38] covered the period from January 2015 to December 2017, while our study spanned from January 2017 to February 2021. Additionally, Audeh et al [38] included only 1 web forum specialized in thyroid issues, whereas we incorporated this specific forum along with 16 others. The main ADEs associated with Levothyrox in our study align with those found by Audeh et al [38] on similar data, albeit without using the interface. In our study, the 10 most frequent symptoms were pain, tiredness, faintness, hypothyroidism, dizziness, insomnia, palpitations, hyperthyroidism, malignant tumor, and anxiety. By contrast, Audeh et al [38] reported tiredness, weight gain, pain, ganglions, hot flush, chilly, inflammation, faintness, weight loss, and discomfort.

Furthermore, the PHARES pipeline surpasses previous efforts, particularly regarding several criteria. These include the annotation tool, where only 4 pipelines were identified using a French annotator tool. In terms of available statistics, only 1 pipeline met both criteria we identified. Regarding signal detection, among the 3 criteria identified, 5 pipelines matched with only 1, while the remaining 5 matched with none.

In the use case, a notable increase in the number of ADEs associated with Levothyrox was detected using the change-point analysis method a few months after the introduction of the new formula in March 2017, specifically in August 2017. This surge coincided with the initial declaration to the pharmacovigilance network and a petition initiated by patients to reintroduce the former formula in June 2017. We compared these findings with results from a pharmacovigilance study based on spontaneous reporting. Out of 1554 notifications spontaneously addressed by patients to the Pharmacovigilance Center of Nice from January 1, 2017, to December 31, 2017, 1372 were related to the new formula of Levothyrox, representing 7342 ADEs. Our comparison with these data clarified our findings. The 10 most frequently reported ADEs in these notifications closely resembled our own results [37]. These were asthenia, headache, dizziness, hair loss, insomnia, cramps, weight gain, nausea, muscle pain, and irritability. Consequently, our results demonstrate coherence with the existing literature. This study illustrates the feasibility of identifying the date of significant events related to a drug. However, it is noteworthy that the detection of such events is not necessarily expedited through social media compared with the traditional pharmacovigilance system.

### Limitations

The method used in our annotation process was integrated at an early stage during the pipeline’s design. Regarding the identification of drugs and symptoms, our annotation process exhibited the following performances: precision=0.926, recall=0.845, and *F*_1_-score=0.886 [20]. Similarly, for discerning the relationship between the drug and the ADEs, the performances were precision=0.683, recall=0.956, and *F*_1_-score=0.797 [20]. This study marked the inaugural publication on using NLP methods to identify ADEs in French-language web forums. The annotation process was thus developed using contemporary state-of-the-art methodologies at the time. However, it would now stand to gain from the integration of more recent NLP algorithms for named entity recognition [8,23,24]. These newer algorithms offer comparable performances while effectively handling more complex data, thereby enhancing the efficacy of NLP analysis. However, because of our emphasis on the genericity of the approach and the interoperability between the different modules rather than solely focusing on the performance of each module, we opted not to use these algorithms. Nevertheless, contemporary state-of-the-art methods for annotating ADEs from social media posts encompass convolutional neural networks trained on top of pretrained word vectors for sentence-level classification [24] and transformers using the bidirectional encoder representations from transformers (BERT) language model [39]. Hussain et al [25] introduced a multitask neural network based on BERT with hyperparameter optimization capable of sentence classification and named entity recognition. This model achieved performances of precision=0.840, recall=0.861, and *F*_1_-score=0.896 on the Twitter (X)-TwiMed data set. Additionally, Magge et al [8] presented a pipeline consisting of 3 BERT neural networks designed to classify sentences, extract named entities, and normalize those entities to their respective MedDRA concepts. The performances of this model were as follows: precision=0.82, recall=0.76, and *F*_1_-score=0.78 on the SMM4H-2020 data set (Twitter/X). Thanks to our modular design, it will be straightforward to substitute our current annotation process with an enhanced model in the future.

Several limitations should be acknowledged for future work. First, the scraper relies on the HTML structure of web forums, necessitating updates to its configuration files if a forum alters its page design. Additionally, our interface lacks the capability to incorporate alternate identifiers for drugs or ADEs. For instance, patients may commonly refer to the drug “baclofen” as “baclo” on social media platforms. Consequently, the number of posts pertaining to a drug or ADE could potentially be underestimated.

Forums must be selected before query execution to mitigate calculation time. However, selecting forums based on the presence of information related to a particular drug or ADE can introduce bias into signal detection methods, particularly in disproportionality analysis, where the drug-ADE pair may be overrepresented. Another limitation in qualitative analysis of posts is the inability of users to edit annotations or record typical pharmacovigilance qualitative data.

The assumption that all drugs mentioned in a post were consumed simultaneously by the user, as applied in the logistic regression–based method, introduces an evident bias.

One limitation associated with the use of social media data pertains to fraudulent posts. The pseudonymity inherent in these platforms provides malevolent individuals with the opportunity to disseminate false rumors. Additionally, patients might post identical or similar messages across multiple discussion boards, or even multiple times on the same board. Thus, it is crucial to consider these factors to mitigate biases in signal detection.

### Perspectives

In the short to medium term, our objectives are updating the annotation module to enhance accuracy, improving the qualitative analysis by enabling users to edit and correct annotations, and expanding the range of signal detection methods available in the statistics module.

This method could indeed be beneficial for identifying potential drug misuse and unknown ADEs [40]. By categorizing pathological terms found in web forums based on their presence in the summary of product characteristics, we can distinguish between indications, known ADEs, and potential instances of drug misuse or unexpected ADEs. However, it is important to note that considering all pathological terms found in the summary of product characteristics as indications might obscure cases of drug inefficiency. Therefore, a nuanced approach is necessary to ensure comprehensive and accurate analysis.

We next tested our pipeline from the perspective of end users. However, the hypothesis was only partially confirmed, indicating the need for further studies. These studies should include evaluations with ergonomic criteria.

In the long term, our vision is to expand this tool to encompass other languages and themes beyond pharmacovigilance. This includes areas such as drug misuse, the consumption of food supplements, and the use of illegal drugs. French web forums dedicated to recreational drug use already exist, providing a valuable source of data for such endeavors.

### Conclusions

Our hypothesis focused on the challenge encountered by regulatory agencies in using social media, primarily because of the lack of appropriate decision-making tools. To tackle this challenge, we devised a pipeline consisting of 4 editable modules aimed at effectively analyzing health-related French web forums for pharmacovigilance purposes. Using this pipeline and its user-friendly interface, we successfully demonstrated the feasibility of conducting quantitative analyses without the need for coding. This approach yielded coherent results and holds the potential to reveal new insights about drugs.

A practical implication of our pipeline is its potential application in health surveillance by regulatory agencies such as the ANSM or pharmaceutical companies. It can be instrumental in detecting issues related to drug safety and efficacy in real time. Furthermore, research teams can leverage this tool to retrospectively analyze events and gain valuable insights into pharmacovigilance trends.

## Appendix

Multimedia Appendix 1 Vigi4Med Scraper structure, PHARES database structure, example of data representation, and source and evolution over time of web forum posts. PHARES: Pharmacovigilance in Social Networks.

## Abbreviations

ADE: adverse drug event
ANSM: Agence nationale de sécurité du médicament et des produits de santé
ATC: Anatomical Therapeutic Classification
BERT: Bidirectional Encoder Representations from Transformer
CSV: comma-separated values
CUSUM: Cumulative Sum
EMA: European Medicines Agency
FDA: Food and Drug Administration
FPVD: French Pharmacovigilance Database
GDPR: General Data Protection Regulation
HAS: French National Health Authority
MedDRA: Medical Dictionary for Regulatory Activities Terminology
NLP: natural language processing
PHARES: Pharmacovigilance in Social Networks
PREDOSE: Prescription Drug Abuse Online Surveillance and Epidemiology
PRR: proportional reporting ratio
PT: preferred term
RDF: resource description framework
ROR: reporting odds ratio
WEB-RADR: Recognizing Adverse Drug Reactions

## Data Availability

Our data were extracted from web forums that do not allow data sharing. Thus, as we are not the owners of the data we cannot make the data available. The scrapper we developed to extract these data is open source and can be used to extract data from web forum posts. The tool as well as full documentation (in English and French) of the code and configuration file are available online [41].
