# Supplementary material for: Monitoring Adverse Drug Events in Web Forums: Evaluation of a Pipeline and Use Case Study
Source: J Med Internet Res. 2024 Jun 18;26:e46176. doi: 10.2196/46176 (PMC11220433; doi:10.2196/46176)
Supplement: Multimedia Appendix 1 [file jmir_v26i1e46176_app1.docx]

# Figures Appendix

**Paper:** Monitoring adverse drug events in web forums: Evaluation of a pipeline that enables final users to interact with a graphical user interface and use case with Levothyrox® new formula in France

**Authors:** Pierre Karapetiantz, Bissan Audeh, Akram Redjdal, Teophile Tiffet, Cédric Bousquet, Marie-Christine Jaulent

**Submission Number:** ms #46176


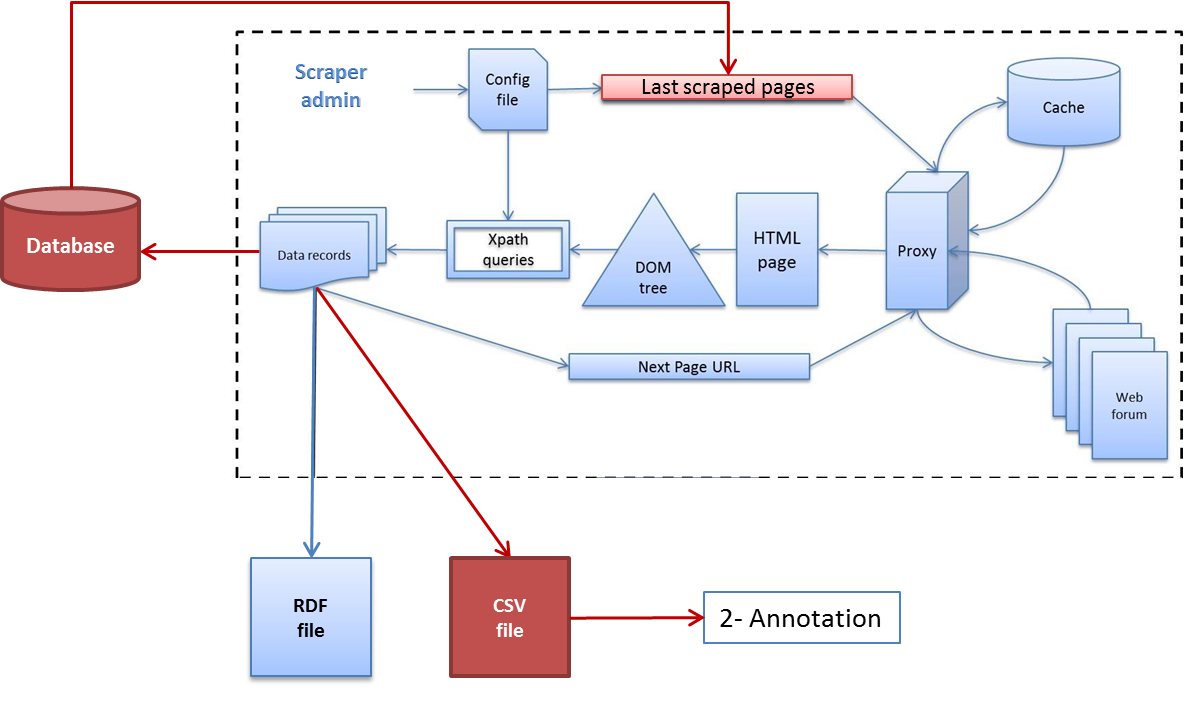


***Supplementary* *Figure 1****. Vigi4Med Scraper’ structure (blue elements), PHARES adaptation (red elements).*


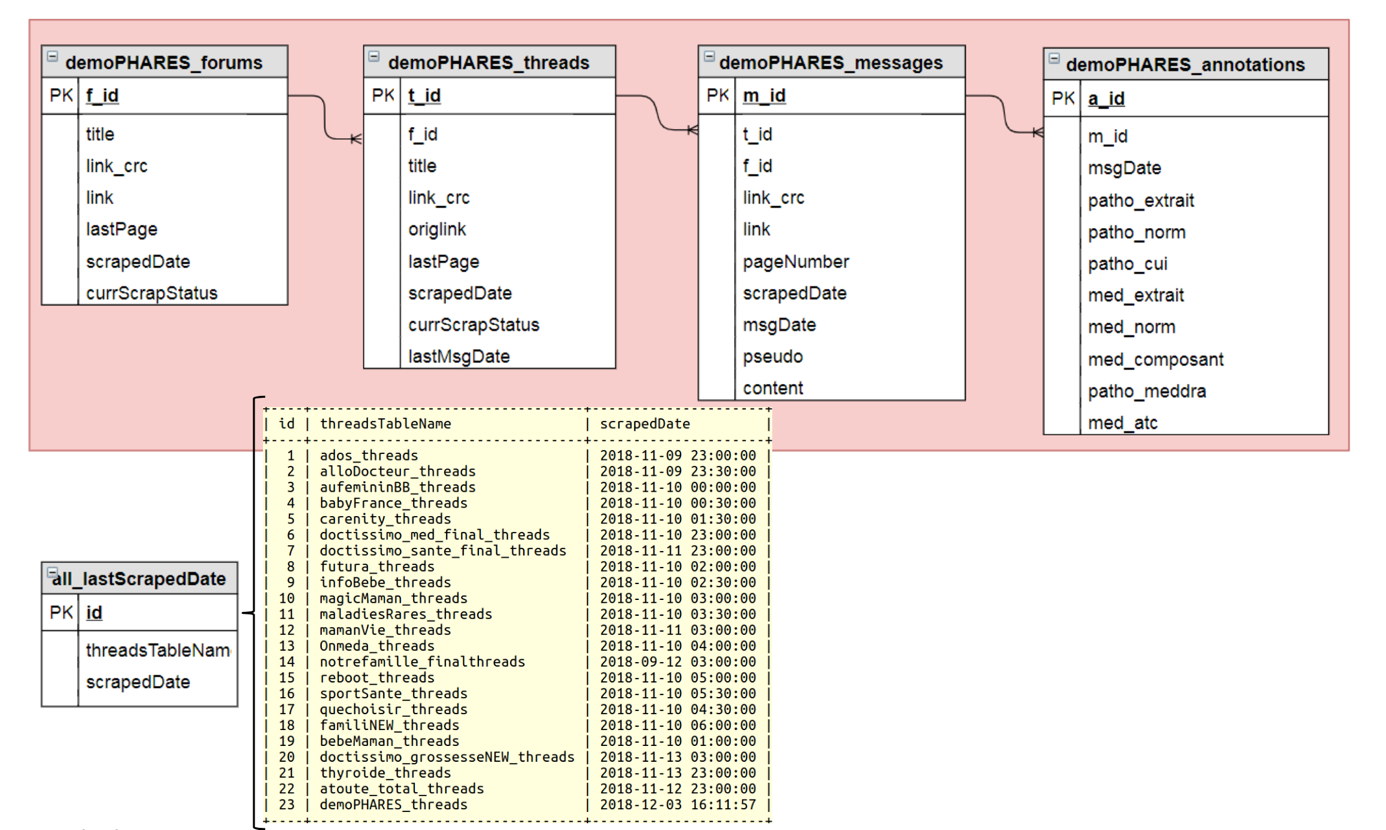


***Supplementary* *Figure 2****. Structure of PHARES database*


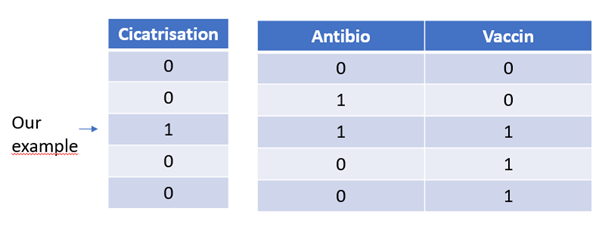


**Supplementary Figure 3**. Example of data representation for logistic regression, data composed of 5 posts (the 3rd being the post in Figure 5). “Cicatrisation” vector represents the absence/presence of the mention of the ADE “wound healing” in the post. The Matrix represents the absence/presence of the mention of the drugs “Antibiotic” and “Vaccine” in the post, 0 represents the absence and 1 the presence


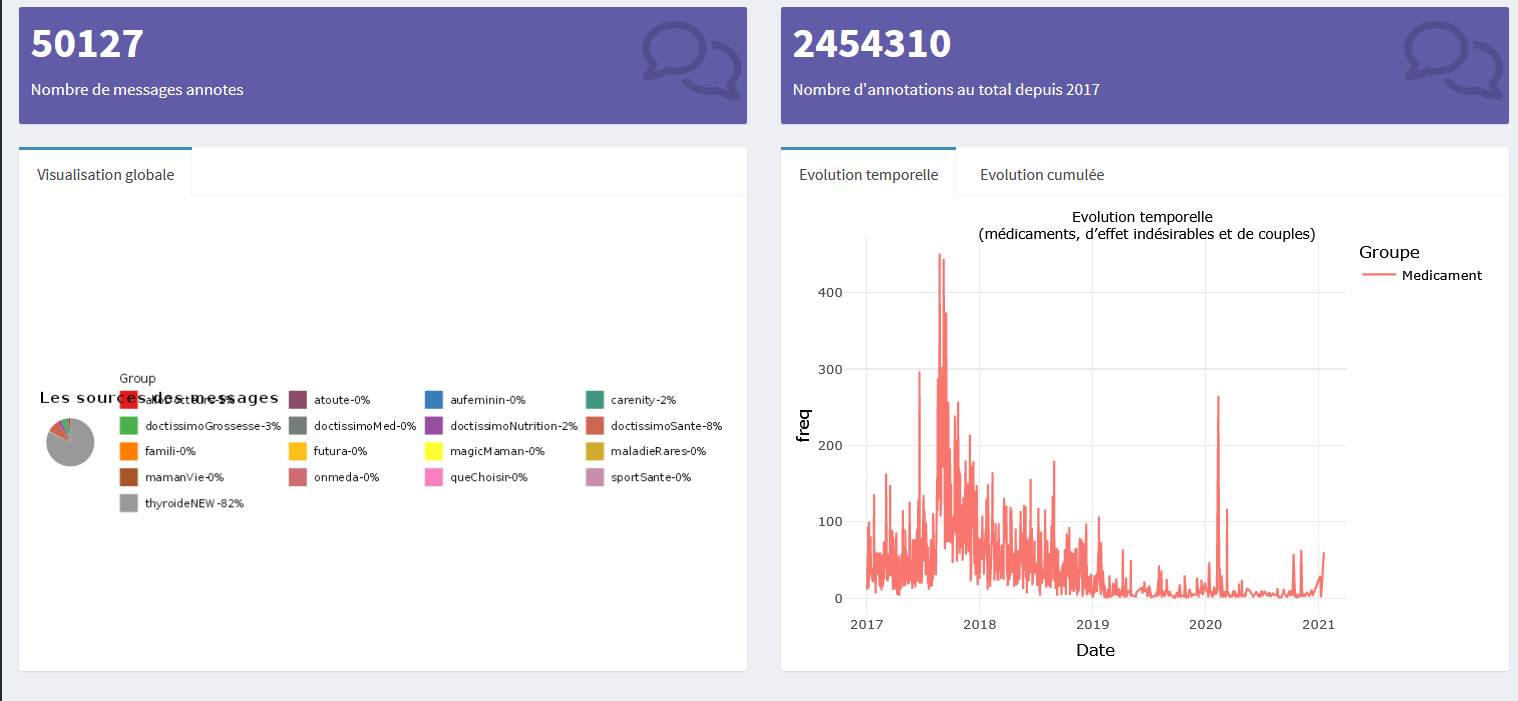


**Supplementary figure 4**. Source and evolution over time of 11,340 French web forums’ posts concerning levothyrox® from 01/01/2017 to 28/02/2021


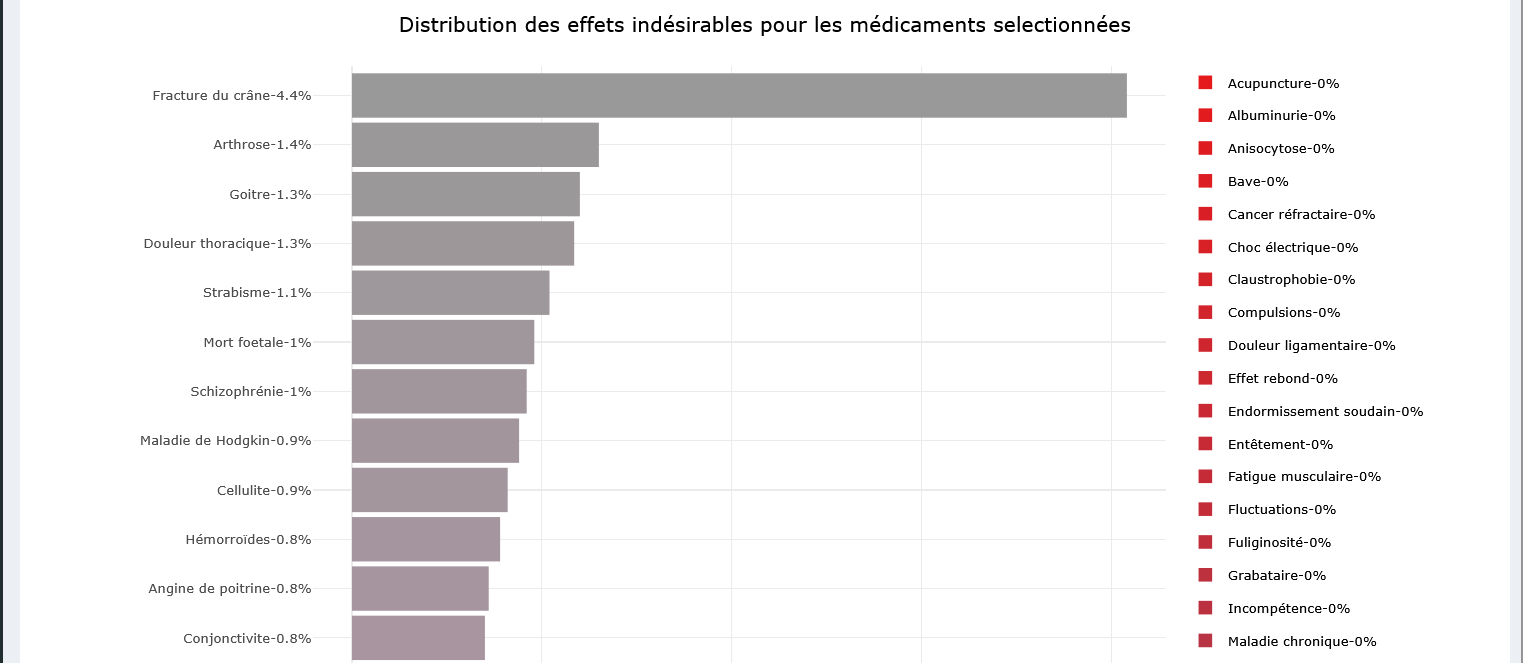


**Supplementary figure 5**. ADEs found with levothyrox® in French web forums’ posts between 01/01/2017 and 28/02/2021’s PT distribution


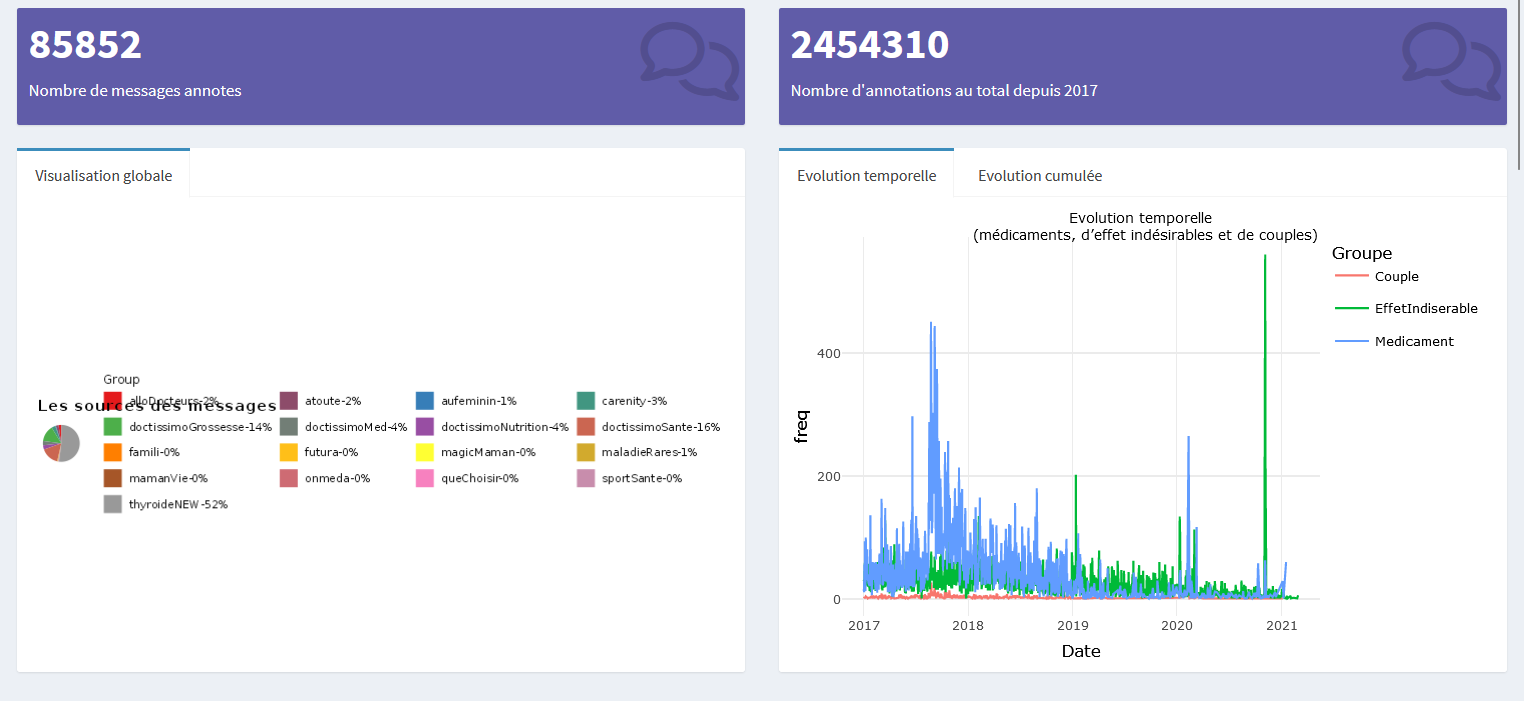


**Supplementary figure 6**. Source and evolution over time of French web forums’ posts concerning levothyrox® and tiredness from 01/01/2017 to 28/02/2021
